# Predicting post-laminoplasty kyphosis in cervical spondylotic myelopathy patients without preoperative kyphosis: a retrospective study

**DOI:** 10.1186/s12891-023-06650-0

**Published:** 2023-06-27

**Authors:** Yiyuan Yang, Ruomu Qu, Zexiang Liu, Zhuo Chen, Yunxia Wu, Hongling Chu, Zhongjun Liu, Xiaoguang Liu, Liang Jiang

**Affiliations:** 1 Department of Orthopaedics, Peking University Third Hospital, Beijing, China; 2 Engineering Research Center of Bone and Joint Precision Medicine, Ministry of Education, Beijing, China; 3 Beijing Key Laboratory of Spinal Disease Research, Beijing, China; 4 Research Center of Clinical Epidemiology, Peking University Third Hospital, Beijing, China

**Keywords:** Cervical spondylotic myelopathy, Laminoplasty, Postoperative kyphosis, C2-7 extension Cobb angle, C2-7 Cobb angle, Predictor

## Abstract

**Background:**

This study aimed to determine potential risk factors for post-laminoplasty kyphosis and the effect of postoperative kyphosis on neurologic function recovery.

**Methods:**

A total of 266 patients with cervical spondylotic myelopathy (CSM) underwent traditional cervical laminoplasty with a minimum of a 12-month follow-up period. The patients were divided into non-kyphosis (NK group) and kyphosis (K group) groups based on the postoperative C2-7 Cobb angle. Clinical and radiological measurements were collected preoperatively and at the final follow-up.

**Results:**

Of the 266 patients, 26 (9.77%) developed postoperative kyphosis at the final follow-up. The postoperative Japanese Orthopedic Association score did not differ significantly between the NK and K groups (*P* > 0.05). The postoperative numeric rating scale (NRS) also showed no significant difference between the NK and K groups; however, postoperative NRS improved better than the preoperative values in the NK group (*P* < 0.001). Multivariate analysis revealed that the preoperative C2-7 extension Cobb angle and C2-7 Cobb angle were independent predictors of post-laminoplasty kyphosis. Cut-off values for predicting postoperative kyphosis were a C2-7 extension Cobb angle of 18.00° and a C2-7 Cobb angle of 9.30°.

**Conclusions:**

Low preoperative C2-7 extension Cobb angle and C2-7 Cobb angle may be associated with post-laminoplasty kyphosis in CSM patients without preoperative kyphosis. The cut-off value of the C2-7 extension Cobb angle and C2-7 Cobb angle were 18.00° and 9.30°, respectively.

## Background

Cervical spondylotic myelopathy (CSM) is one of the most prevalent diseases that leads to neurological dysfunction due to cervical spinal cord compression [1]. Laminoplasty (LP) and posterior cervical decompression and fusion (PCDF) are the two most popular posterior procedures for CSM [2, 3]. Laminoplasty is widely used in treating multi-level CSM due to its satisfactory clinical outcome [4–6]. Maintaining sufficient cervical lordosis is critical in achieving and maintaining indirect decompression by the posterior drift of the spinal cord [7]. Postoperative kyphosis will negatively affect the decompression effect and the recovery of neurologic function [8, 9]. PCDF has the benefit of reducing postoperative segmental instability and the incidence of kyphosis. However, PCDF could reduce cervical motion. It is essential to identify potential factors that cause post-laminoplasty kyphosis, especially in patients without preoperative kyphosis. Furthermore, many radiological parameters have been considered as potential predictors of postoperative kyphosis, including the T1 slope and C2-7 Cobb angle; however, its pathogenic mechanism has not been clarified. Recently, cervical extension function has received more attention because it may be associated with the constriction reservoir of the cervical posterior muscular-ligament complex (PMLC) [10]. Therefore, we hypothesized that poor cervical extension function might be related to postoperative kyphosis.

This study aimed to determine potential risk factors for post-laminoplasty kyphosis and the effect of postoperative kyphosis on neurologic function recovery.

## Methods

### Patient population

We retrospectively included 266 consecutive patients with cervical spondylotic myelopathy who underwent laminoplasty performed by our spine team between January 2015 and January 2022. The inclusion criteria were a diagnosis of CSM, cord compression on magnetic resonance imaging (MRI), and a minimum of 1 year of follow-up. Exclusion criteria included preoperative cervical kyphosis, previous spinal surgeries, congenital cervical deformity, history of cervical fracture and/or infection, and history of ankylosing spondylitis and/or rheumatoid arthritis. We also excluded patients whose C7 vertebral bodies could not be identified on lateral radiographs, which made it difficult to measure the C2-7 Cobb angle. The ethics committee of our hospital approved this study. The requirement for informed consent was waived due to the retrospective nature of our study.

### Operative procedure

All patients underwent unilateral expansive open-door laminoplasty performed by seven spine surgeons with at least five years of experience in spine surgery. The posterior cervical spine was exposed via a longitudinal midline approach. We then performed LP using the Hirabayashi method [4]. We resected the ventral cortex on the open side and removed the cephalad and caudal ends of the laminar doors. Then we placed anchor sutures around the base of the spinous processes to prevent lamina closure. All patients were allowed to get out of bed on the first day after surgery with a cervical collar for two weeks.

### Clinical and radiological outcome evaluations

Demographic data were retrospectively collected from the electronic medical records. Clinical outcomes were evaluated using the numeric rating scale (NRS), the Japanese Orthopedic Association (JOA) score, and its recovery rate.

**Fig. 1:**
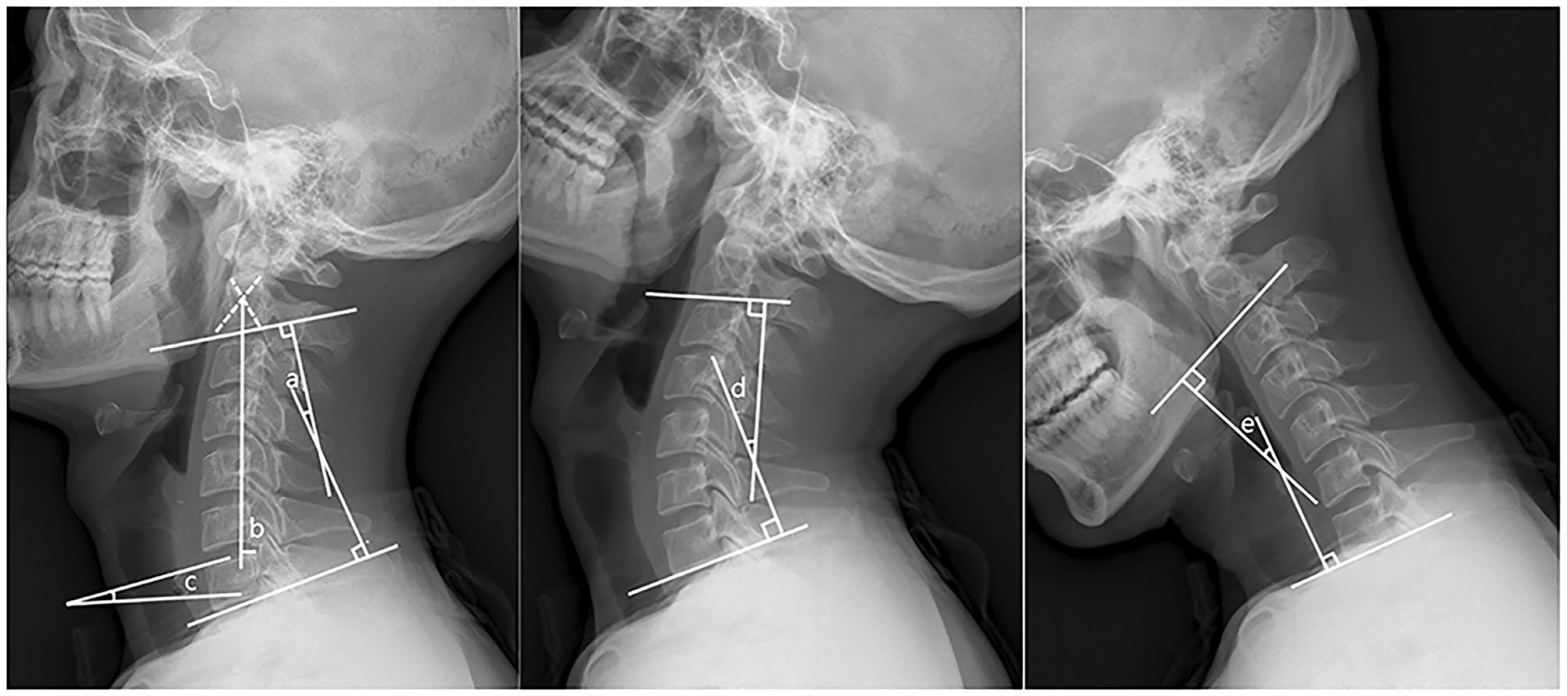
Measurements of cervical radiological parameters. (**a**) C2-7 Cobb angle; (**b**) C2-7 SVA; (**c**) C7 slope; (**d**) C2-7 extension Cobb angle; (**e**) C2-7 flexion Cobb angle

Radiological parameters were measured using neutral and flexion-extension radiographs preoperatively and at follow-up. The cervical curvature and range of motion (ROM) were evaluated according to the Cobb angle method [11]. The C2-7 sagittal vertical axis (SVA) was measured as the total distance from the plumb line of the center of the C2 vertebra to the posterosuperior corner of the C7 vertebra. The C7 slope was defined as the angle between the horizontal line and the upper endplate of C7 (Fig. 1). The postoperative kyphotic deformity was defined as a C2-7 Cobb angle of < 0° postoperatively. Patients were divided into the subgroups of non-kyphosis (NK group) and kyphosis (K group) according to the postoperative C2-7 Cobb angle.

### Statistical analysis

All statistical analyses were performed using Statistical Package for the Social Sciences (version 23.0; SPSS Inc., Chicago, IL, USA). All values are expressed as mean ± standard deviation. Between-group differences were analyzed using the Mann-Whitney U test, unpaired *t*-test for continuous variables and Fisher’s exact test for categorical variables. Changes in parameters (from the preoperative evaluation to the final follow-up visit) were compared using the Wilcoxon rank sum test or the paired *t*-test. Multivariate logistic regression analysis was performed. A receiver operating characteristic (ROC) curve was drawn to determine the cut-off values of preoperative parameters for predicting post-laminoplasty kyphosis. Statistical significance was set at P < 0.05.

## Results

### Population details

Among the 266 patients included in our study were 176 men and 90 women, with a mean age of 55.44 ± 10.53 (30–78) years. The average preoperative JOA score was 12.76 ± 2.06 in all patients. The mean operative time was 114.65 ± 37.52 (53–238) min, and the mean intraoperative blood loss was 168.63 ± 115.10 (50-1000) ml. The mean follow-up was 32.14 ± 7.23 (12–36) months (Table 1). The mean postoperative JOA score was 15.08 ± 1.74, and the mean JOA recovery rate was 55.01%. The mean neck pain NRS was 2.13 ± 2.37 before surgery and 1.12 ± 1.72 after the surgery, while the mean arm pain NRS was 1.52 ± 2.40 preoperatively and 0.93 ± 1.63 postoperatively.

**Table 1:** Demographic characteristics and complications

Variables	All(N = 266)	Group NK(N = 240)	Group K(N = 26)	*P* value
Age (years)	55.44 ± 10.53	55.33 ± 10.65	56.54 ± 9.54	0.854
Sex (males: females)	176:90	156:84	20:6	0.222
BMI (kg/m^2^)	25.24 ± 3.29	25.14 ± 3.27	26.20 ± 3.34	0.105
Duration of symptom (months)	45.71 ± 78.70	43.67 ± 76.01	64.48 ± 100.11	0.962
Operation time (minutes)	114.65 ± 37.52	114.04 ± 36.15	120.35 ± 48.87	0.979
Operation blood loss (ml)	168.63 ± 115.10	167.61 ± 116.31	178.08 ± 104.88	0.408
Follow-up time (months)	32.14 ± 7.23	32.18 ± 7.21	31.85 ± 7.55	0.823
Incidence of complications(%)				
C5 nerve palsy	4.89	4.51	0.38	1.000
CSF leak	1.12	1.12	0.00	1.000
Axial pain	3.00	2.63	0.37	0.566
SSI	1.12	1.12	0.00	1.000

Values given are mean ± SD unless otherwise specified

BMI indicates body mass index; SD, standard deviation; CSF, cerebrospinal fluid; SSI, surgical site infection

There were no significant differences in demographic data between the NK and K groups. Postoperatively, the incidence of C5 nerve palsy, cerebrospinal fluid (CSF) leak, axial pain, or surgical site infection (SSI) showed no significant differences between the NK and K groups. None of the patients underwent revision of the index surgery at the final follow-up (Table 1).

The mean preoperative C2-7 Cobb angle was 12.74°±8.35°, C2-7 extension Cobb angle was 26.73°±10.35° and C2-7 flexion Cobb angle was − 17.96°±10.56° in all patients. The mean preoperative C7 slope and C2-7 SVA were 21.59°±7.10° and 20.25 ± 10.32 mm, respectively (Table 2). The mean postoperative C2-7 Cobb angle was 13.19°±10.50°, C2-7 extension Cobb angle was 23.45°±10.02° and C2-7 flexion Cobb angle was − 7.91°±11.87°. The mean postoperative C7 slope and C2-7 SVA were 22.35°±7.34° and 21.72 ± 11.52 mm, respectively.

**Table 2:** Preoperative radiological parameters

Variables	ALL (N = 266)	Group NK (N = 240)	Group K (N = 26)	*P* value
C2-7 Cobb angle (°)	12.74 ± 8.35	13.51 ± 8.36	5.69 ± 3.88	< 0.001*
C2-7 extension Cobb angle (°)	26.73 ± 10.35	27.90 ± 9.99	15.95 ± 7.01	< 0.001*
C2-7 flexion Cobb angle (°)	-17.96 ± 10.56	-17.57 ± 10.69	-21.49 ± 8.73	0.048*
ROM (°)	43.37 ± 13.88	43.67 ± 14.02	40.55 ± 12.46	0.276
C2-7 SVA (mm)	20.25 ± 10.32	19.98 ± 10.41	22.79 ± 9.26	0.097
C7 slope (°)	21.59 ± 7.10	21.88 ± 7.17	18.87 ± 5.85	0.039*

Values given are mean ± SD unless otherwise specified

*Statistically significant values

ROM indicates range of motion; SVA, sagittal vertical axis; SD, standard deviation

### Relationship between preoperative factors and postoperative kyphosis

At the final follow-up, postoperative kyphosis was observed in 26 (9.77%) patients, including twenty males and six females. The preoperative C2-7 Cobb angle was 13.51°±8.36° in the NK group and 5.69°±3.88° in the K group (*P* < 0.001). Preoperative C2-7 extension Cobb angle was 27.90°±9.99° in the NK group and 15.95°±7.01° in the K group (*P* < 0.001). Preoperative C2-7 flexion Cobb angle was − 17.57°±10.69° in the NK group and − 21.49°±8.73° in the K group (*P* < 0.05). The NK group had a significantly greater C7 slope than the K group (*P* < 0.05). However, there was no significantly different in the preoperative ROM (*P* > 0.05) and C2-7 SVA (*P* > 0.05) between the two groups (Table 2). Multivariate analysis showed that the preoperative C2-7 extension Cobb angle (Odds ratio = 0.895, *P* < 0.001) and C2-7 Cobb angle (Odds ratio = 0.874, *P* < 0.05) were associated with post-laminoplasty kyphosis (Table 3).

**Table 3:** Logistic regression analysis for cervical postoperative kyphosis

Variables	Partial regression coefficient	SE	Wald value	*P* value	OR
C2-7 Cobb angle (°)	-0.135	0.063	4.559	0.033*	0.874
C2-7 extension Cobb angle (°)	-0.111	0.032	12.275	< 0.001*	0.895
C2-7 flexion Cobb angle (°)	-0.002	0.029	0.004	0.952	0.998
C7 slope (°)	-0.004	0.051	0.006	0.939	0.996
C2-7 SVA (mm)	0.021	0.030	0.483	0.487	1.021

*Statistically significant values

SE indicates standard error; OR, Odds ratio

### ROC analysis of potential parameters to predict postoperative kyphosis

**Fig. 2:**
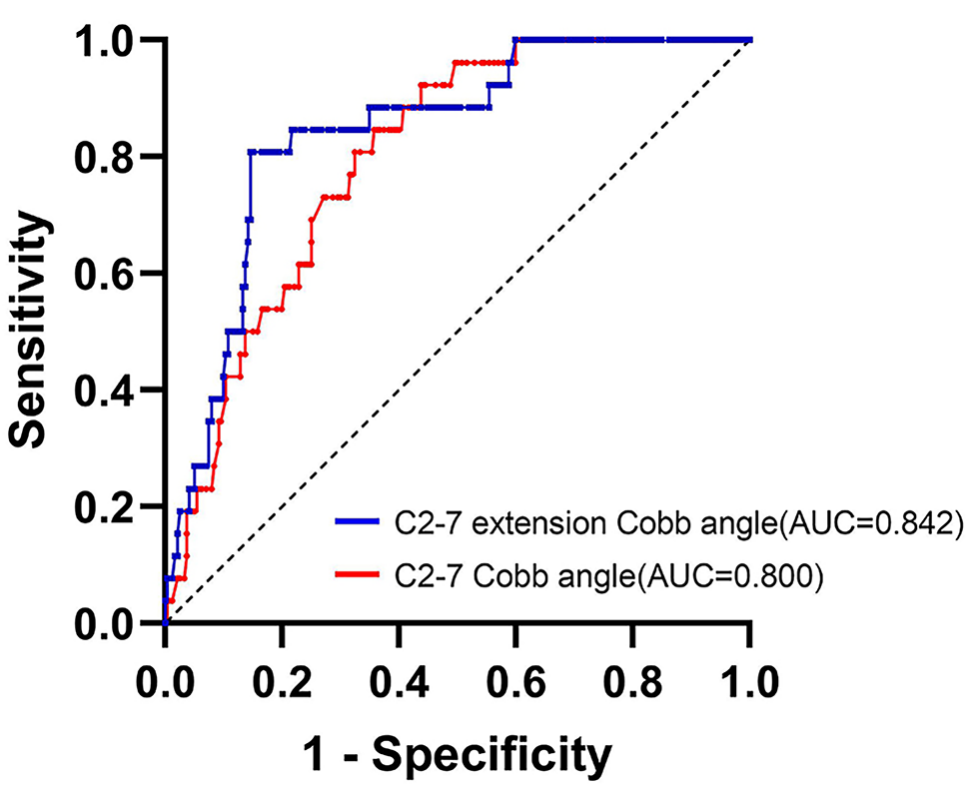
The ROC curve of C2-7 extension Cobb angle and C2-7 Cobb angle for the prediction of post-laminoplasty kyphosis

**Fig. 3:**
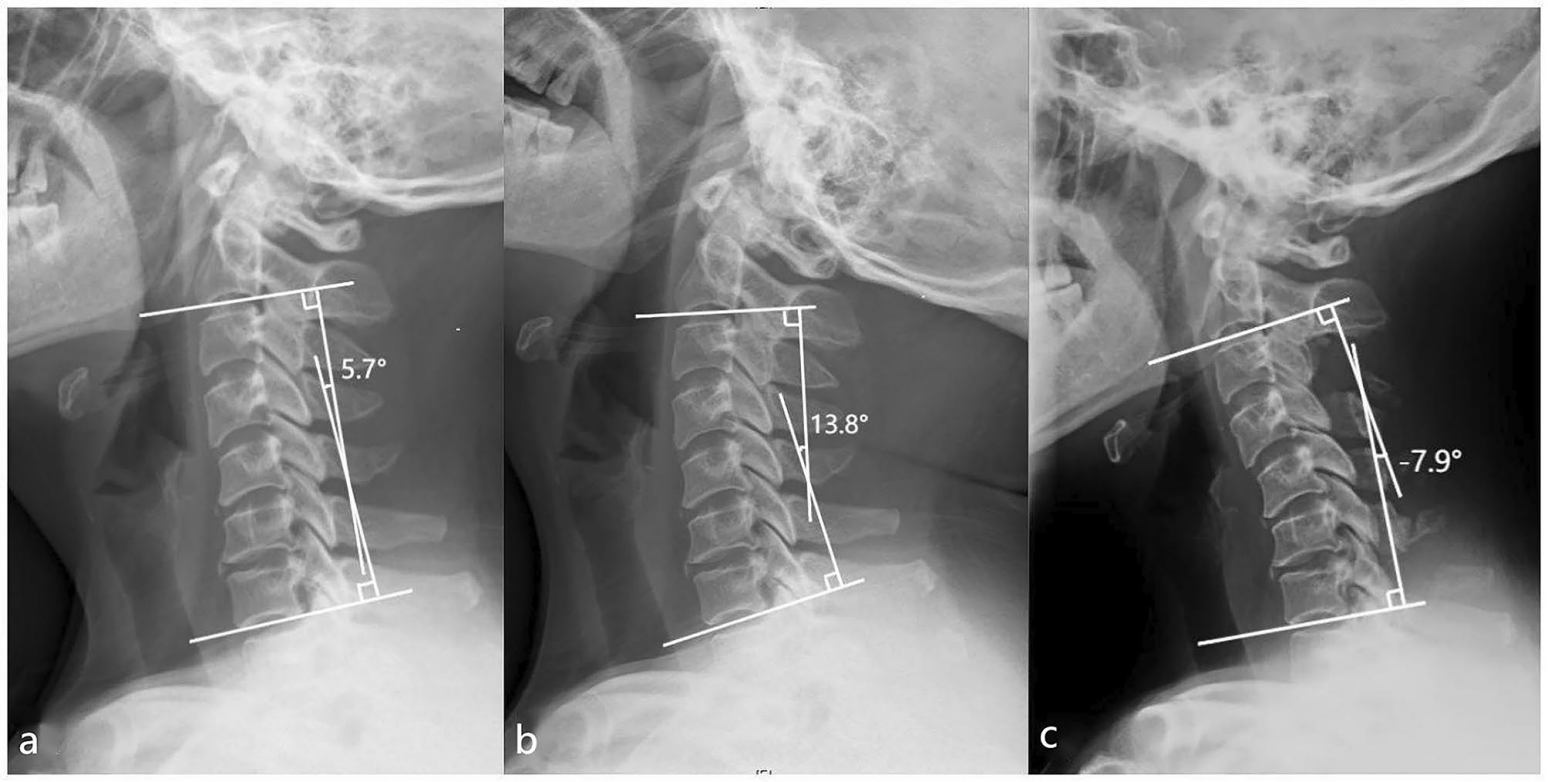
Case of a 47-year-old woman. (**a**) The preoperative radiograph showed that the C2-7 Cobb angle was 5.7°. (**b**) The preoperative radiograph showed that the C2-7 extension Cobb angle was 13.8°. (**c**) Postoperative radiograph at the final follow-up showed that the C2-7 Cobb angle was − 7.9°

ROC analysis demonstrated that a cut-off value of 18.00° of C2-7 extension Cobb angle was corelated to 80.77% sensitivity and 85.42% specificity, and that of 9.30° of C2-7 Cobb angle was corelated to 84.62% sensitivity and 64.17% specificity for predicting post-laminoplasty kyphosis. The area under the curve of the C2-7 extension Cobb angle and C2-7 Cobb angle were 0.842 and 0.800, respectively (Fig. 2). Among 108 patients with a preoperative C2-7 Cobb angle ≤ 9.30°, postoperative kyphosis occurred in 21 (19.44%). Among 56 patients with a preoperative C2-7 extension Cobb angle ≤ 18.00°, postoperative kyphosis occurred in 21 (37.50%). Of the 48 patients with the preoperative C2-7 Cobb angle ≤ 9.30° and the preoperative C2-7 extension Cobb angle ≤ 18.00°, 20 developed post-laminoplasty kyphosis (41.67%) (Fig. 3).

### Neurological function recovery

There was no significant difference in the postoperative arm pain NRS and neck pain NRS between the NK and K groups (*P* > 0.05). However, postoperative arm pain NRS improved better than the preoperative value in the NK group (*P* < 0.001). In addition, the postoperative JOA scores did not differ significantly between the two groups (*P* > 0.05). The JOA recovery rates in the NK and K groups were 54.98% and 55.28%, respectively (*P* > 0.05) (Table 4).

**Table 4:** Clinical parameter assessments

Variables		Group NK (N = 240)	Group K (N = 26)	*P* value
NRS (neck pain)	Pre	2.08 ± 2.37	2.58 ± 2.44	0.301
	Post	1.09 ± 1.71	1.38 ± 1.90	0.412
	*P* value	< 0.001*	0.044*	
NRS (arm pain)	Pre	1.45 ± 2.36	2.15 ± 2.77	0.140
	Post	0.90 ± 1.60	1.23 ± 1.88	0.377
	*P* value	< 0.001*	0.131	
JOA score	Pre	12.69 ± 2.11	13.42 ± 1.38	0.135
	Post	15.05 ± 1.77	15.39 ± 1.43	0.352
	*P* value	< 0.001*	< 0.001*	
JOA recovery rate (%)		54.98 ± 31.00	55.28 ± 29.26	0.962

Values given are mean ± SD unless otherwise specified

*Statistically significant values

NRS indicates numeric rating scales; JOA, the Japanese Orthopaedic Assossciation; Pre, preoperative; Post, postoperative; SD, standard deviation

## Discussion

Many studies have reported postoperative kyphosis after laminoplasty, while posterior cervical decompression and fusion could maintain cervical lordosis and prevent postoperative kyphosis [8, 9, 12]. The purpose of this study was to determine the relationship between preoperative parameters and postoperative kyphosis after laminoplasty for CSM patients without preoperative kyphosis. In this study, 9.77% of patients without preoperative kyphosis developed postoperative kyphosis, and multivariate analysis demonstrated that the preoperative C2-7 extension Cobb angle and C2-7 Cobb angle were associated with post-laminoplasty kyphosis. In addition, preoperative C2-7 flexion Cobb angle, C2-7 Cobb angle, C2-7 extension Cobb angle, and C7 slope showed statistically significant differences between the NK and K groups in univariate analysis. In previous studies, the incidence of post-laminoplasty kyphosis was reported to be approximately 10-15% [8, 13, 14], and postoperative kyphosis may occur due to multiple factors, such as C2-7 SVA, C2-7 Cobb angle, surgical methods, and cervical extension function [15–18].

Our results showed that the preoperative C2-7 extension Cobb angle was associated with postoperative kyphosis, which is consistent with the studies of Suk et al. and Lee et al. [8, 10]. Suk et al. demonstrated that patients with a greater Cobb angle during flexion than during extension were more prone to develop post-laminoplasty kyphosis. Lee et al. also pointed out that postoperative kyphosis may be associated with cervical extension function. Unlike Lee’s study, we used the C2-7 extension Cobb angle to manifest the function of PMLC. The PMLC is vital for maintaining cervical sagittal balance and horizontal gaze. When patients undergo laminoplasty, the PMLC will inevitably be damaged to some extent, resulting in a decline in muscle contractility. Therefore, patients with smaller C2-7 extension Cobb angles, meaning less PMLC function, are more likely to develop post-laminoplasty kyphosis. In this study, a preoperative C2-7 extension Cobb angle of 18.00° was a predictive factor for post-laminoplasty kyphosis in patients with CSM. The risk of postoperative kyphosis in the group with a C2-7 extension Cobb angle ≤ 18.0° was higher than that in the group with a C2-7 extension Cobb angle > 18.0° (37.5% vs. 2.4%). These results suggested that laminoplasty might be carefully selected for patients with smaller C2-7 extension Cobb angles, and PCDF might be an alternative method for those patients. In addition, preoperative neck muscle isometric contraction training may be beneficial for the maintenance of cervical lordosis.

In a retrospective cohort study, Michino et al. [19] reported a preoperative lordotic angle of < 7° may predict post-laminoplasty kyphosis. Choi et al. [15] suggested that the cut-off C2-7 Cobb angle of 8.5° was a predictor for predicting postoperative kyphosis. In the current study, a preoperative C2-7 Cobb angle of 9.3° was a predictive factor for post-laminoplasty kyphosis in patients with CSM. The incidence of postoperative kyphosis in the group with a C2-7 Cobb angle ≤ 9.3° was higher than that in the group with a C2-7 Cobb angle > 9.3° (19.4% vs. 3.2%).

Because of shoulder interference, the T1 vertebral body is not always visible on cervical radiographs. Tamai et al. [20] reported that the C7 slope was closely associated with the T1 slope and showed a similar relationship with other radiological parameters as the T1 slope. In this study, the C7 slope was used instead of the T1 slope. The preoperative C7 slope was not significantly associated with post-laminoplasty kyphosis in multivariate analysis. Cho et al. [21] also suggested that postoperative kyphosis was not associated with the preoperative T1 slope. However, Kim et al. [22] suggested that a high T1 slope might be a predictive factor for post-laminoplasty kyphotic alignment changes. In the study by Kim et al., the mean value of the T1 slope was 30.9°±3.9° in the high T1 slope group. In contrast, the mean C7 slope in this study was 21.6°±7.1°, which was lower than that of the high T1 slope group in Kim et al. ’s study. Differences in the composition of the patient population may account for this finding. Therefore, further studies are needed to determine the role of the T1 slope in postoperative kyphosis.

This study observed no statistically significant difference in the preoperative C2-7 SVA between the NK and K groups. Furthermore, it is generally accepted that preoperative C2-7 SVA is positively correlated with loss of cervical lordosis [23]. Sakai et al. [12] proposed that a preoperative center of gravity of the head (CGH)-C7 SVA > 42 mm was a predictive factor for post-laminoplasty kyphosis. One possible reason for this inconsistency is that most of our cases had low preoperative C2-7 SVA, which was not high enough to cause a statistical difference.

The general view is that kyphotic changes may negatively impact clinical outcomes. Baba et al. [7] pointed out that maintaining cervical lordosis was key to obtaining better clinical results due to the mechanism of spinal cord drift. Sakaura et al. [24] also found poor clinical prognoses in patients with kyphosis after cervical surgery. In our study, although there was no significant difference in the postoperative NRS and JOA scores between the two groups, the postoperative NRS improved better than the preoperative values in the NK group. This finding indicates that postoperative kyphosis may affect the improvement of pain symptoms.

Our study has several limitations, including its retrospective nature. In addition, we included some patients in 2022, and a minimum follow-up period of one year may not be sufficient. Furthermore, the relationship between some thoraco-lumbar or spino-pelvic parameters of the whole spine and postoperative kyphosis has not been evaluated.

## Conclusions

Low preoperative C2-7 extension Cobb angle and C2-7 Cobb angle may be associated with post-laminoplasty kyphosis in CSM patients without preoperative kyphosis. The cut-off value of the C2-7 extension Cobb angle and C2-7 Cobb angle were 18.00° and 9.30°, respectively. In cases with low preoperative C2-7 extension Cobb angle and C2-7 Cobb angle, posterior cervical decompression and fusion might be an alternative method.

## Data Availability

The datasets used and/or analysed during the current study are available from the corresponding author on reasonable request.

## Abbreviations

CSM: Cervical spondylotic myelopathy
LP: Laminoplasty
PCDF: Posterior cervical decompression and fusion
PMLC: Posterior muscular-ligament complex
MRI: Magnetic resonance imaging
NRS: Numeric rating scale
JOA: Japanese Orthopedic Association score
ROM: Range of motion
SVA: Sagittal vertical axis
NK: Non-kyphosis
K: Kyphosis
ROC: Receiver operating characteristic curve
CSF: Cerebrospinal fluid
SSI: Surgical site infection
